# Genetic characterization of norovirus GII.4 variants circulating in Canada using a metagenomic technique

**DOI:** 10.1186/s12879-018-3419-8

**Published:** 2018-10-17

**Authors:** Nicholas Petronella, Jennifer Ronholm, Menka Suresh, Jennifer Harlow, Oksana Mykytczuk, Nathalie Corneau, Sabah Bidawid, Neda Nasheri

**Affiliations:** 1 Biostatistics and Modeling Division, Bureau of Food Surveillance and Science Integration, Food Directorate, Health Canada Ottawa, Ottawa, ON Canada; 2 Department of Food Science and Agricultural Chemistry, Faculty of Agricultural and Environmental Sciences, Macdonald Campus, McGill University, Montreal, QC Canada; 3 Department of Animal Sciences, Faculty of Agricultural and Environmental Sciences, Macdonald Campus, McGill University, Montreal, QC Canada; 4 National Food Virology Reference Centre, Bureau of Microbial Hazards, Food Directorate, Health Canada 251 Sir Frederick Banting Driveway, Ottawa, ON K1A 0K9 Canada

**Keywords:** Norovirus, Next-generation sequencing, Metagenomics, Recombination, Antigenic drift, Co-infection

## Abstract

**Background:**

Human norovirus is the leading cause of viral gastroenteritis globally, and the GII.4 has been the most predominant genotype for decades. This genotype has numerous variants that have caused repeated epidemics worldwide. However, the molecular evolutionary signatures among the GII.4 variants have not been elucidated throughout the viral genome.

**Method:**

A metagenomic, next-generation sequencing method, based on Illumina RNA-Seq, was applied to determine norovirus sequences from clinical samples.

**Results:**

Herein, the obtained deep-sequencing data was employed to analyze full-genomic sequences from GII.4 variants prevailing in Canada from 2012 to 2016. Phylogenetic analysis demonstrated that the majority of these sequences belong to New Orleans 2009 and Sydney 2012 strains, and a recombinant sequence was also identified. Genome-wide similarity analyses implied that while the capsid gene is highly diverse among the isolates, the viral protease and polymerase genes remain relatively conserved. Numerous amino acid substitutions were observed at each putative antigenic epitope of the VP1 protein, whereas few amino acid changes were identified in the polymerase protein. Co-infection with other enteric RNA viruses was investigated and the astrovirus genome was identified in one of the samples.

**Conclusions:**

Overall this study demonstrated the application of whole genome sequencing as an important tool in molecular characterization of noroviruses.

**Electronic supplementary material:**

The online version of this article (10.1186/s12879-018-3419-8) contains supplementary material, which is available to authorized users.

## Introduction

Norovirus (NoV) is a major cause of acute gastroenteritis worldwide, being responsible for sporadic and outbreak cases in various epidemiological settings [1, 2]. To date, there is no licensed antiviral therapy or vaccine available for the treatment or prevention of NoV infections [3, 4]. In the absence of a robust and readily available cell culture system, most of our understanding regarding NoV transmission, evolution, and molecular characteristics has been inferred from the analyses of epidemiological and clinical data [5].

Based on genetic diversity NoVs are classified into 7 genogroups (I–VII). Only genogroups I, II and IV have been found to infect humans. NoV genogroups are further categorized into 38 genotypes [6]. Genogroup II genotype 4 (GII.4) is the most prevalent, comprising many variants which have caused 62% to 80% of all NoV outbreaks globally since the mid-1990s [7, 8]. NoV GII.4 has evolved rapidly during the past 4 decades [9] resulting in new genetic clusters every 2–5 years [10, 11]. While some GII.4 variants such as Cairo 2007, Asia 2003 and Japan 2008 caused local epidemics, variants such as US95/96 1995, Farmington Hills 2002, Hunter 2004, Den Haag 2006b, New Orleans 2009 and Sydney 2012 led to global NoV pandemics [11–14].

Noroviruses are single-stranded, positive sense RNA viruses that belong to the family *Caliciviridae*. The genome is approximately 7.6 kb and contains 3 open reading frames (ORFs). ORF1 encodes a ~ 1700 amino acid polyprotein that is cleaved into 6 non-structural proteins: the p48 protein, an N-terminal protein of unknown function; the 2C-like helicase protein; a 3A-like protein; the VPg protein, a viral genome-linked protein; a 3C-like protease; and the RNA-dependent RNA polymerase (RdRp) [15]. ORF2 encodes the VP1 major capsid protein, which contains two domains: the shell (S) domain and the protruding (P) domain. The P domain of VP1 is further divided into two subdomains: P1 and P2. P2 is the most variable and exposed region of the VP1 protein, which contains antigenic epitopes (A-E) and sites for histo-blood group antigens (HBGAs) binding [16–18]. ORF3 encodes VP2, which is a small basic structural protein [19].

Prior to the advent of next generation sequencing (NGS) technologies, Sanger sequencing was the method of choice for analyzing viral samples, and Sanger sequencing still remains the gold standard for many clinical, environmental, and food-related applications. For characterization of infections associated with NoV, certain regions of the capsid or the polymerase genes are amplified and subjected to Sanger sequencing. While Sanger sequencing is suitable for routine laboratory testing and genotyping, this approach requires the use of standard primers for both amplification and sequencing. The use of standardized primers introduces amplification biases in favor of dominant variants. In addition, less abundant mutations are not detected since base calling methods currently have a 20% detection threshold [20]. Despite not being detected through standard methods, low-frequency mutations in viral populations are associated with drug resistance and strain emergence [21–25]. Also low frequency variants have been employed to elucidate transmission directions in viral infections [21, 22]. Therefore, dependant on the application, studying the genetic diversity of the viral quasispecies can be more informative than focusing on the dominant variants that appear in consensus sequences [20]. Full-length genomic sequences are also required for proper epidemiological analysis and efficient source attribution during sporadic or outbreak infections.

In the present study, we employed next-generation sequencing to expand our knowledge regarding the genetic diversity of GII.4 strains circulating in Canada during a 5-year period, between 2012 and 2016, with a particular focus on GII.4 Sydney 2012 variants. We also analyzed amino acid variations in the major capsid protein and the polymerase protein. Finally, we used the metagenomics data generated by our whole genome sequencing (WGS) approach to identify co-infections with other enteric viruses in the studied samples, and identified that one patient may have had an astrovirus co-infection.

## Methods

### Sample collection and preparation

Fifty-two NoV GII.4 positive fecal samples that were submitted to the National Food Virology Reference Centre at Health Canada and Viral Diseases Division at Public Health Agency of Canada, between 2012 and 2016, were chosen for this study. Samples were collected from five Canadian provinces (Ontario, Newfoundland and Labrador, Nova Scotia, Saskatchewan, and Alberta). Sample preparation, RNA extraction, and amplification were performed as described previously [22]. Sanger sequencing was performed to verify the presence of NoV GII.4. Viral loads were determined by droplet digital PCR (Bio-Rad, Hercules, California, USA) using the conditions that were described previously [22, 26]. A total of 44 samples had viral loads higher than 250 genome copies/μl, and were selected for deep-sequencing using the Illumina MiSeq platform.

### Library preparation and Illumina sequencing

The quality and quantity of extracted RNA was examined using Agilent RNA 6000 Pico Assay Kit and Protocol (Agilent Technologies, Santa Clara, California, USA). Ethanol precipitation of RNA was performed prior to proceeding to TruSeq Stranded mRNA (Illumina, San Diego, California, USA) sample preparation. Library preparation was performed as described previously [22]. The prepared cDNA library was subjected to paired end sequencing on a MiSeq Reagent Kit v3 (150-cycle).

### De novo assembly and analysis

Reads were assembled de novo using SPAdes version 3.9.0. Contigs containing NoV sequence data were identified using BLASTn against a continually updated in-house database, as described previously [22], comprised of all NoV sequences available from NCBI. Once NoV contigs were identified and extracted, PROKKA v1.11 was used to identify all the ORFs.

In order to identify the total number of NoV reads per sample in addition to coverage, all reads were subject to a reference guided assembly using SMALT v0.7.4 (https://sourceforge.net/projects/smalt/).

The sequencing reads (SRA) for each sample were deposited in GenBank under the accession numbers SRR6743837 to SRR6743880.

### Construction of phylogenetic trees

**Fig. 1:**
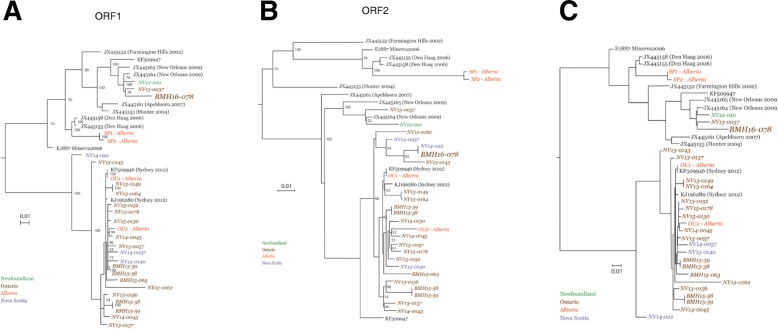
Phylogenetic analysis of individual ORFs. Consensus sequences obtained in this study along with certain full-length Canadian sequences and reference sequences were aligned and phylogenetic trees were constructed for ORF1 (**a**), ORF2 (**b**) and ORF3 (**c**) using the Maximum Likelihood method. The robustness of the phylogeny was assessed through bootstrap analysis of 1000 pseudo-replicates. Sequences in brown are isolated from Ontario, orange from Alberta, blue for Nova Scotia, green from Newfoundland and Labrador. The recombinant sequence is shown in bold. The sequences generated in this study are italicized

**Fig. 2:**
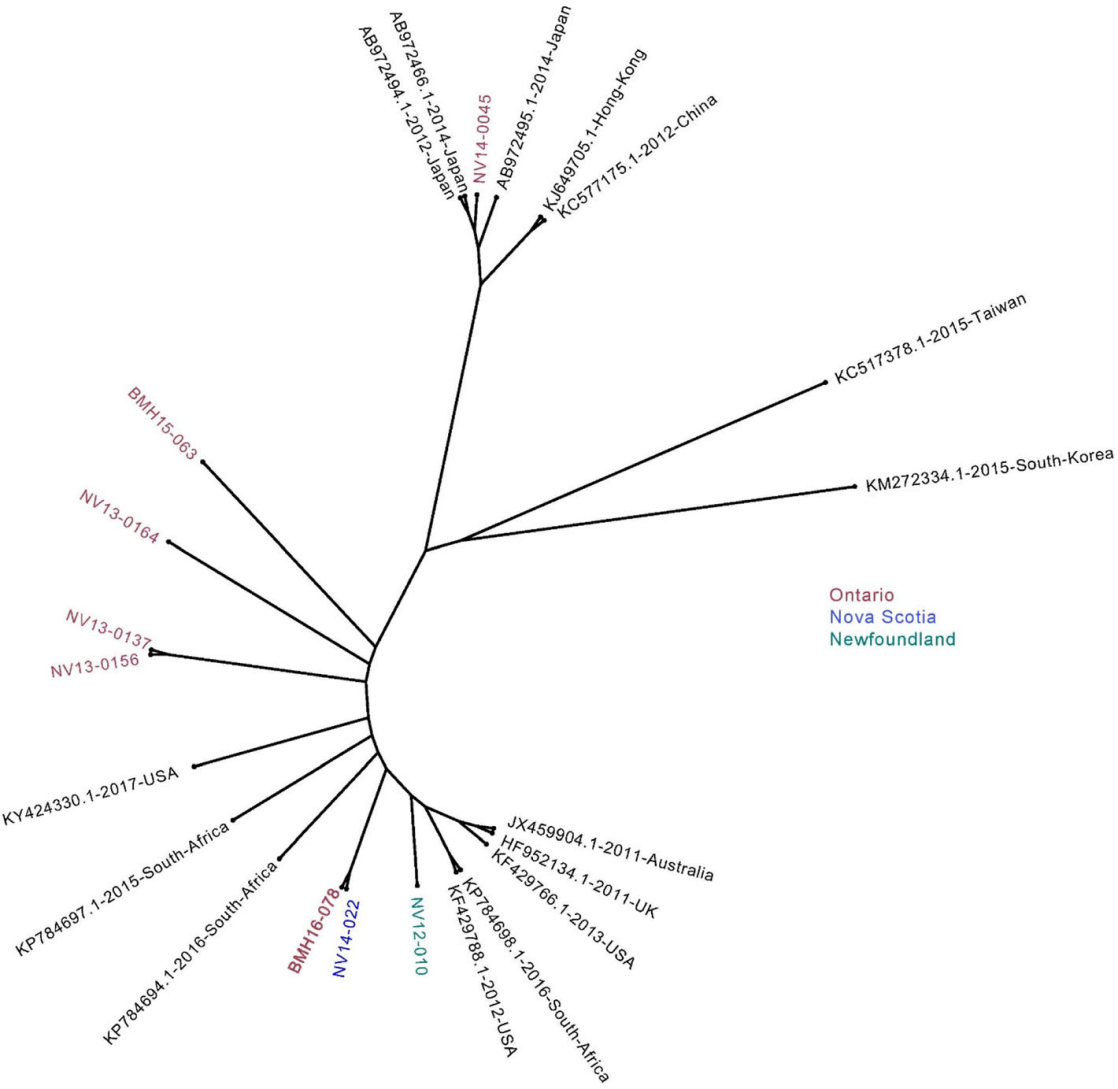
Phylogenetic analysis of the full-length genome sequences. A phylogenetic tree was constructed using certain full-length genome sequences obtained in this study and several full-length sequences from different geographical regions using the Maximum Likelihood method. Sequences in red are isolated from Ontario, blue for Nova Scotia, green from Newfoundland. The recombinant sequence is shown in bold

Phylogenetic trees consisted of either aligned ORFs found in Fig. 1 or whole NoV genomes found in Fig. 2. Nucleotide sequences were aligned using MUSCLE [27] and phylogenetic trees were constructed from resulting alignments with RAxML v8.1.1 implementing a GTR Gamma nucleotide substitution model [28] for 1000 bootstrap replicates.

### Recombination analysis

Potential recombination within the complete genome sequences was screened using seven methods (RDP, GENECONV, MaxChi, Bootscan, Chimera, SiScan, and 3Seq) implemented in the Recombination Detection Program version 4.46 (RDP4) [29]. The breakpoints were also defined by RDP4. Similarity between the recombinants and their possible major and minor parents was estimated using BootScan, embedded in RDP4 [28]. SimPlot [30] was used to visualize the relationships between the recombinant and its possible parents. The annotation of the nucleotide is based upon NCBI nucleotide accession number JX445164.

### Similarity analysis

Similarities between the aligned Sydney 2012 nucleotide sequences were visualized using the SimPlot program [30]. The similarity was examined using a window size of 200 nucleotides in length (nt) and a step size of 20 nt in the full-length NoV genomes.

## Results

### Overall sequencing outcome

A total of 24 Giga base-pairs (Gb) raw sequencing data from 4 paired end Illumina MiSeq runs (6 Gb on average) was generated. The sequencing reads were assembled into contigs via de novo genome assembly. From the 44 samples that were subjected to Illumina MiSeq sequencing, near full-genome sequences (coverage > 90%) were obtained from 19 samples and partial sequences were acquired from the rest of the samples, with a median read depth of 376-fold for the full genome sequences (Additional file 1: Table S1). Despite a large sequencing depth allocated to each sample (on average: 2.75 million reads), only a relatively small proportion of the obtained reads were mapped to NoV genomes in the samples that generated full-genome sequences (on average 2.5% mapped to NoV genome). Genome-wide depth of coverage for each genome was examined by measuring the number of reads per position, and as shown in Additional file 1: Figure S1, the depth of coverage was not uniform across the genomes; the 5′ and 3′ ends of the genomes consistently showed lower coverage. This observation has already been reported by others, as well as our group [22, 31, 32], and it has been explained by the inherent difficulty in recovering readable sequences at the ends of DNA fragments from the short sequences produced by Illumina. Consistent with our previous observations and other reports, samples with higher load had better coverage [22, 33].

### Phylogenetic analysis

Nineteen near-full length genomes generated in this study were chosen for further analyses. Four GII.4 Sydney 2012 near full-genome sequences were obtained from our previous work (BMH15–58, BMH15–59, BMH13–38, and BMH13–39) [22], and 4 Canadian GII.4 full genome sequences have already been deposited in GenBank (SP1-Alberta, SP2-Alberta, OU1-Alberta, and OU2-Alberta) [34]. Phylogenetic analysis was performed on these sequences along with a collection of reference sequences representing a variety of NoV GII.4 strains. Three phylogenetic trees were constructed, one for each ORF (Fig. 1a-c). The majority of sequences are most closely related to Sydney 2012 strains (GenBank accession No KF509946 and KJ196280) (Fig. 1), whereas SP1-Alberta and SP2-Alberta cluster with Den Haag 2006 strains (GenBank accession No JX445155 and JX445158), and NV12–010 and NV13–0037 cluster with New Orleans 2009 strains (GenBank accession No JX445164 and JX445165). Evidence indicates that BMH15–58 and BMH15–59, BMH13–38 and BMH13–39, SP1-Alberta and SP2-Alberta, which have high sequence homology, are epidemiologically linked [22, 34]. The NV13–0149 and NV13–0164 sequences also demonstrate significant homology to one another at each ORF (Fig. 1a-c). These samples were collected from the same province within the same calendar year. However, further epidemiological data are needed to confirm whether these cases were linked as well.

The ORF2 of BMH16–078 resembles GII.4 Sydney 2012 (Fig. 1b), while the ORF1 and ORF3 show a higher similarity to New Orleans 2009 sequences (Fig. 1a and c, respectively). This observation is indicative of a recombination event, and therefore, further analysis was performed.

In order to analyze the sequence homology between the sequenced Canadian GII.4 isolates and the variants circulating globally, selected full-length GII.4 sequences from different geographical regions were acquired from GenBank and aligned with some of the sequences obtained in this study. As depicted in Fig. 2, except for NV14–0045, which clusters with the sequences from South East Asia, the rest of the sequences demonstrate homology to the sequences from the United States, South Africa, United Kingdom and Australia.

### Genetic recombination analysis

Genetic recombination is a major driving force in the evolution and emergence of novel GII.4 variants [11, 12, 33, 35]. Genetic recombination occurs when a single cell is co-infected with two NoV variants and, therefore, indicates co-infection of an individual with both variants. Consequently, the detection of a recombination event is important for understanding local and global epidemiology. Since most recombination events between norovirus genomes take place at or near the ORF1/ORF2 or ORF2/ORF3 overlap regions, it is necessary to analyze all three ORFs to identify recombinant viruses [2, 36].

**Fig. 3:**
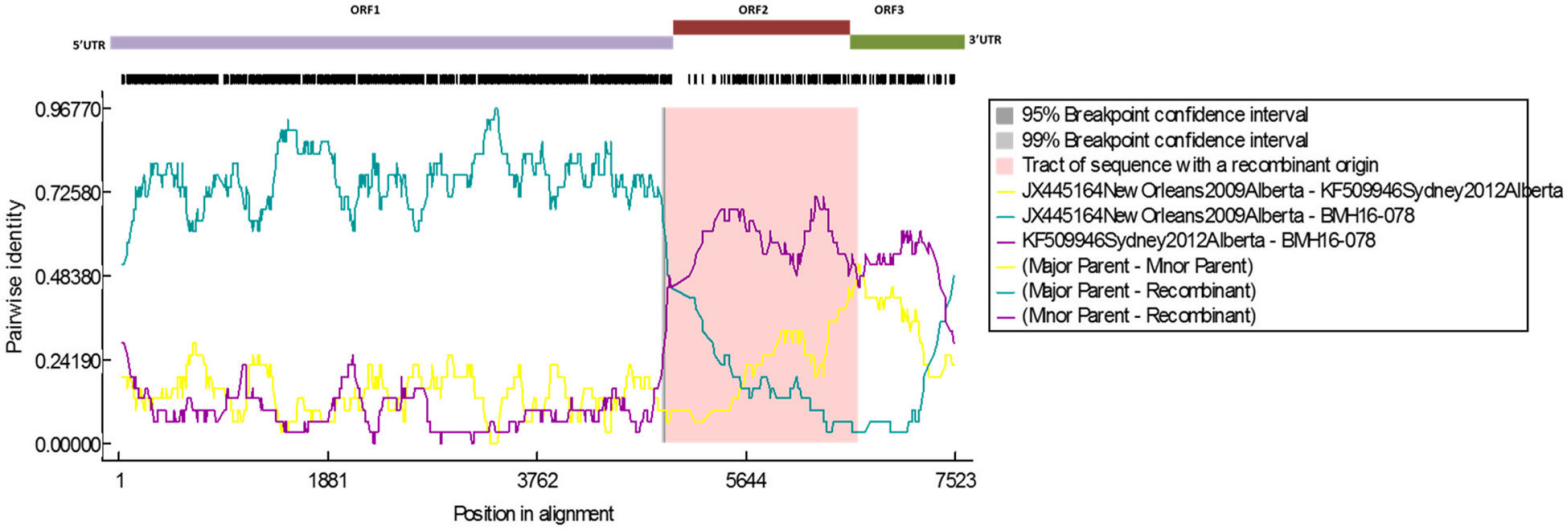
SimPlot analysis of the complete genomic sequence of BMH16–078 recombination. SimPlot was constructed using the RDP4 Software version 4.72 with a slide window width of 200 bp and a step size of 20 bp. At each position of the window, the query sequence was compared to each of the reference strains. The X-axis indicates the nucleotide positions in the multiple alignments of the NoV sequences; and the Y-axis indicates nucleotide identities between the query sequence and the NoV reference strains

The complete genome sequences obtained in this study were analyzed by RDP4 to determine the presence of NoV genomic recombination. As depicted in Fig. 3, BMH16–078 shared a high level of identity in nucleotide sequences in ORF1 and ORF3 with the New Orleans 2009 (JX445164) strains, but in ORF2 with the Sydney 2012 (KF509946) strains. The breakpoints of recombination were located near the ORF1/2 and ORF2/3 overlap regions, hence, creating a recombinant New Orleans 2009 virus with a Sydney 2012 capsid.

### Similarity analysis

**Fig. 4:**
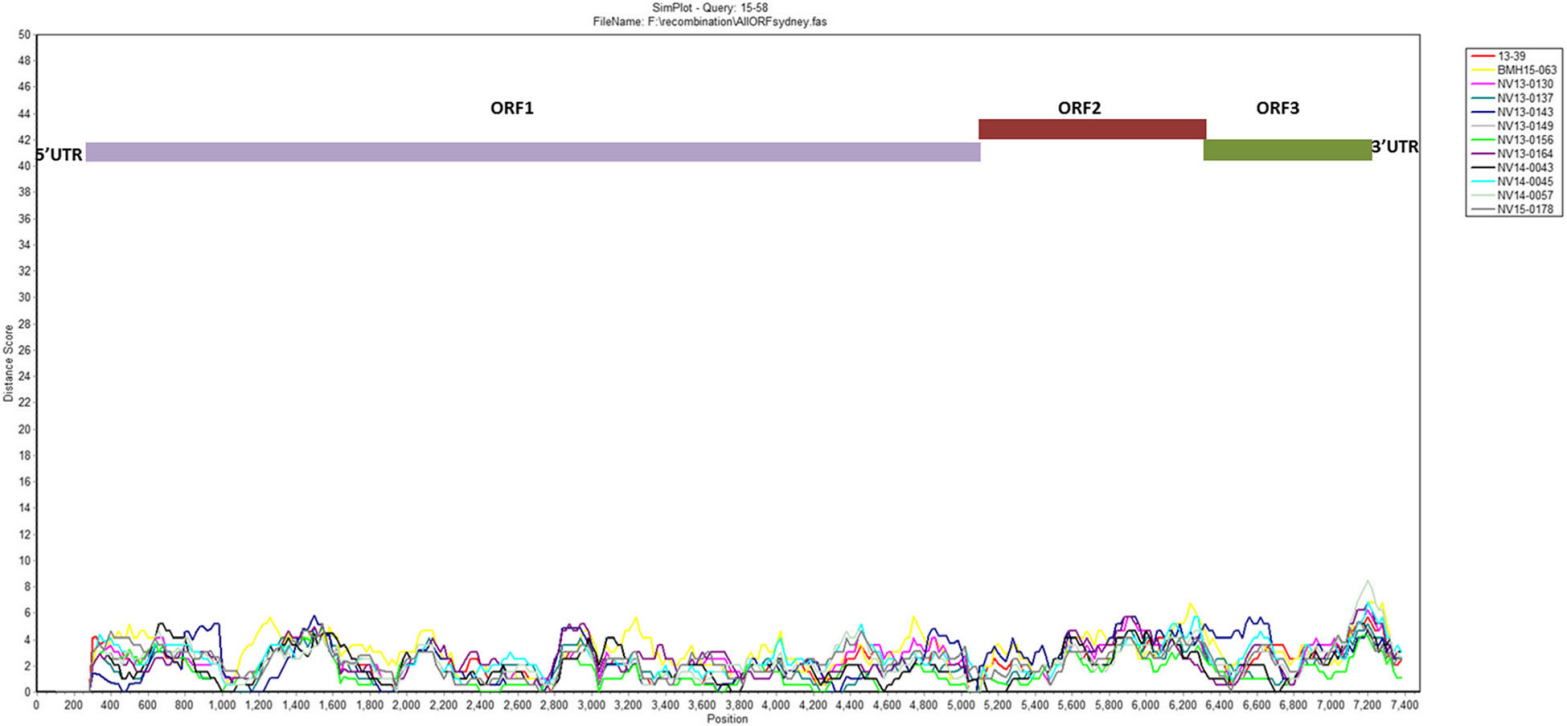
SimPlot analysis of the complete genomic sequences of GII.4 Sydney 2012. SimPlot was constructed using a Simplot software version 3.5 with a slide window width of 200 bp and a step size of 20 bp. At each position of the window, the query sequence was compared to other Sydney 2012 variants sequenced in this study. The X-axis indicates the nucleotide positions in the multiple alignments of the NoV sequences; and the Y-axis indicates nucleotide difference (%) between the query sequence (BMH15–58) and other sequenced Sydney 2012 strains

In order to investigate sequence heterogeneity and identify potential mutation “hot-spots”, full sequences from the Sydney 2012 variants were aligned and nucleotide differences were visualized using SimPlot software. The diversity plot reveals a homogenous distribution of sequence variability in the genome (Fig. 4). The diversity between the genomes increases at the major and minor capsid genes (5085 nt to 6705 nt and 6707 nt to7513nt, respectively), while the regions corresponding to the viral protease and RNA dependent RNA polymerase (RdRP) genes (3029 nt to 3571 nt and 3572 nt to 5101 nt) seem to be more conserved. The average distance score in the hypervariable region of the VP1 gene (5841 nt to 6031 nt) is 3.9% ± 0.45% and for the VP2 gene (7101 nt to 7321 nt) is 4.7% ± 0.75%. While the average distance score for the protease genes and conserved regions of RdRP (3572 nt to 4351 nt are 1.8% ± 0.54% and 1.6% ± 0.37%, respectively (Fig. 4). Furthermore, relatively higher sequence heterogeneity was observed in parts of the p48, NTPase and VPg genes (5–994, 995–2092, 2093–2629, respectively). Altogether, these results suggest that while the capsid proteins are under selective pressure for rapid evolution and diversification, little genetic diversity can be tolerated in the viral protease and RdRP proteins.

### Amino acid variations in the VP1 protein

We performed comprehensive analyses of amino acid changes in the entire VP1 protein for each of the sequences present in our GII.4 alignment, and mapped amino acid substitutions to functional domains plus putative epitopes. Sixty variable sites were detected, representing over 11% of the total VP1 protein of 540 residues. Sixty percent of the variable sites (36 positions) were located in the P2 region of the capsid, with substitutions falling in all 5 recognized blockade epitopes (epitopes A (aa294–298, aa362, aa368), B (aa333, aa382), C (aa340, aa376), D (aa393–395), and E (aa407, aa412–413) [16, 17]. Amino acid variations were identified in 4 out of 5 conformational epitopes (regions 1–5) [9]. However, no substitution was observed in HGBA binding pocket sites I, II, and III (i.e., aa343–347, 374, and 442–443), respectively [37], further validating the conservation of human histo-blood group antigens (HBGAs) binding site among GII.4 variants.

**Fig. 5:**
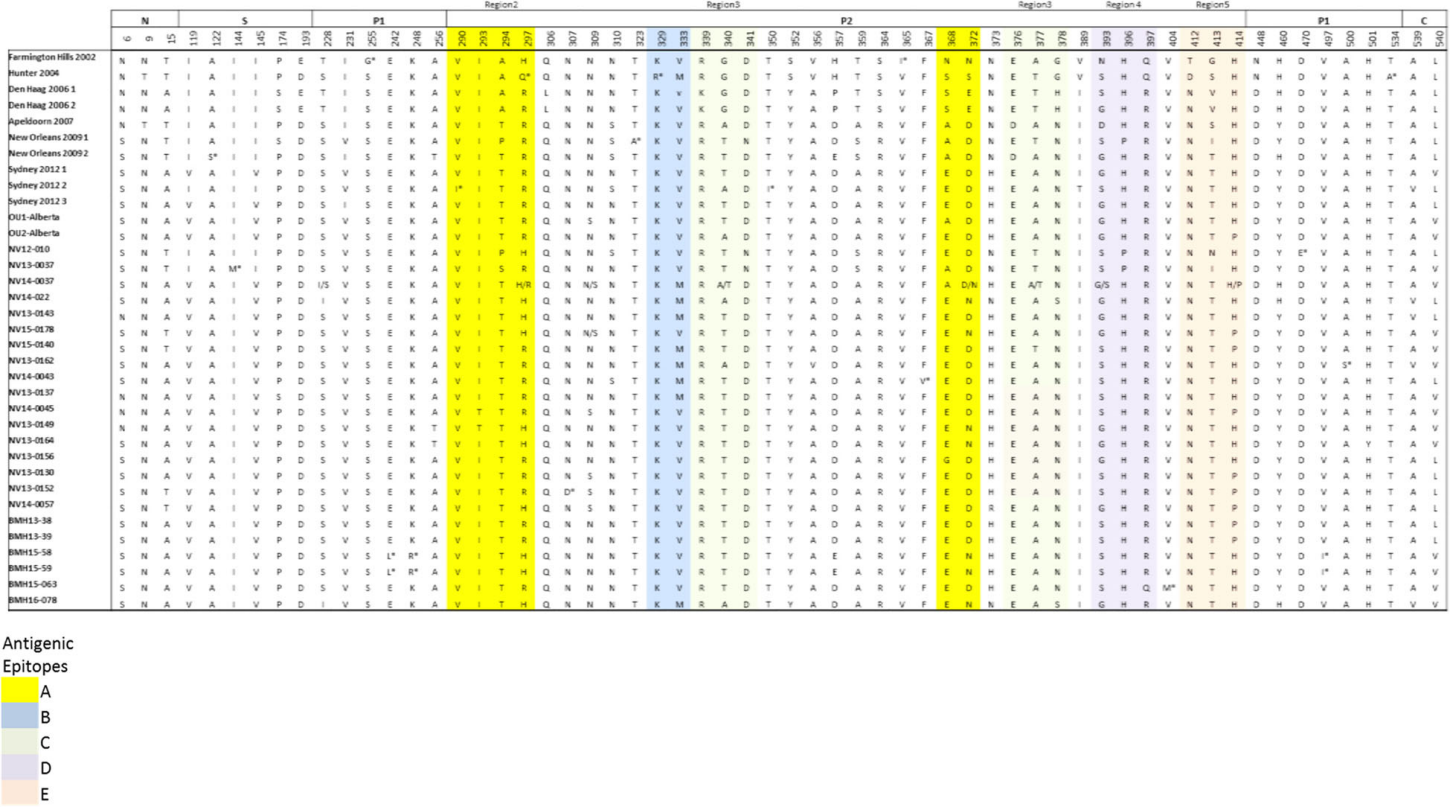
Non-synonymous differences within the structural domains of the capsid protein (VP1, ORF2), which are the N-terminal (N), shell (S), P1, and P2 domains. Individual epitope sites are highlighted in different colors and putative conformational epitopes are shown as regions 2–4. Unique variants are shown by *. Accession numbers for Farmington Hills 2002, Hunter 2004, Den Haag 2006 1, Den Haag 2006 2, Apeldoorn 2007, New Orleans 2009 1, New Orleans 2009 2, Sydney 2012 1, Sydney 2012 2, and Sydney 2012 3 are JX445152, JX445153, JX445158, JX445155, JX445161, JX445164, JX445145, KF509946, KF509947, KJ96280, respectively

Overall, 17 amino acid residues (indicated by an asterisk in the sequence alignment) were present only in one isolate (unique variants). Also, amino acid heterogeneity was observed for 9 sites where two variants for the same location were observed at ratios higher than 30% (Fig. 5).

### Amino acid variations in the polymerase protein

**Fig. 6:**
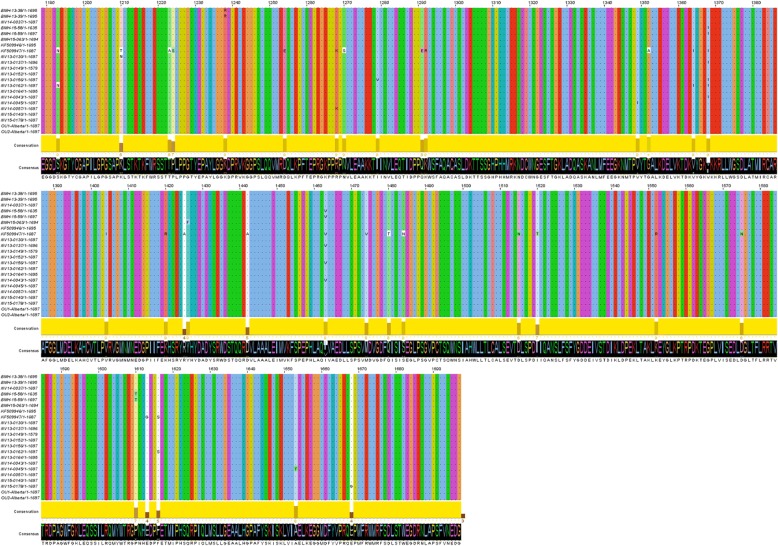
Amino acid variations within the RNA-dependent RNA polymerase (RdRP) protein of the Sydney 2012 sequences. The Jalview histogram below the alignment indicates the conservation of the physico-chemical properties for each column (lower bars with lower numbers, lower conservation; completely conserved columns are in yellow)

The amino acid variations within the polymerase protein of the aligned Sydney 2012 variants were also examined. Overall 33 amino acid changes were identified, which represent 6.4% of the total residues in this protein. The degree of physico-chemical (polarity, hydrophobicity, charge, molecular weight, etc.) conservation was analyzed using Jalview 2.1 software [38] (Fig. 6). No amino acid change was observed within the active site cleft [39–41], and only 5 residues had medium to low conserved physico-chemical properties (5 ≥ Score) [42]. However, in vivo studies would be required to determine whether these amino acid variations have any effect on the polymerase activity.

### Analysis of co-infection

Due to the metagenomics approach that was employed, we were able to explore the possibility of the presence of other pathogenic RNA viruses that may have co-infected each patient. We were especially interested in investigating whether other enteric viruses such as astrovirus, aichi virus, sapovirus, and coxsackievirus B2 would be present in any of the studied samples. For this reason, assembled contigs for each patient were searched against the complete BLAST nucleotide database for the presence of the nearest homologues to these enteric viruses. The BLAST results identified several short contigs for astrovirus in patient NV13–0152 (Additional file 1: Figure S2), which may indicate a co-infection with this virus. This finding is not surprising as human astrovirus is a common cause of pediatric diarrhea worldwide [43], and co-infection with NoV is likely in childcare facilities.

## Discussion

In the field of food safety, quick and accurate detection and characterization of foodborne pathogens is crucial for effective source attribution and risk mitigation. NGS technologies allow for comprehensive investigation of viral genomes without prior knowledge of the target sequences and can sequence full genomes without introducing amplification biases [44]. The sequenced genomes can then be used for epidemiological studies and source tracking. In this study, we employed a metagenomics approach to sequencing NoV genomes directly from clinical samples. The sequence reads generated by this technique can also be mined to investigate the presence of other RNA viruses, pathogenic (co-infections) or non-pathogenic (viral indicators).

Although multiple NoV genotypes co-circulate every season, GII.4 has been the dominant variant worldwide since the early 1990s, and has been responsible for the majority of NoV outbreaks during the last 20 years [2, 14, 42]. In Canada, GII.4 continued to be the most predominant genotype, responsible for 47.6% to 80.2% of all NoV outbreaks [14]. Due to high prevalence and evolution rate of GII.4, we set out to perform genomic characterization of this virus to identify factors that could be associated with its increased epidemic activity.

We have previously demonstrated that the viral titre, and therefore the quantity of viral RNA present in the sample, has a strong effect on the proportion of sequence reads that can be mapped to the NoV genome, and, therefore, on the coverage of the viral genome [22]. Consequently, Illumina sequencing was only performed on 44 samples with viral titres higher than 250 genome copies/μl. Full-genome sequences were obtained from 19 samples, and partial sequences with varying degrees of coverage were retrieved form the remaining 25 samples. For comprehensive analysis of the NoV genome, in this study we only focused on the full-genome sequences whereas the partial sequences will be included in future studies.

The phylogenetic analysis of the obtained genome sequences, along with reference sequences from GenBank for individual ORFs, revealed that most sequences from this study were homologous to Sydney 2012 strains, while the rest showed homology to New Orleans 2009 strains. The timing of sampling also supports our observation; due to its high transmissibility, Sydney 2012 became the predominant strain in Canada in years subsequent to 2012 [14]. We also performed phylogenetic analysis on full-genome sequences and selected sequences that belong to different geographical regions. NV14–0045 that clustered with several sequences from this study showed homology to isolates from South East Asia, while the remaining sequences were homologous with isolates from South Africa and the United States. Due to the lack of epidemiological data, travel cannot be ruled out for NV14–0045 patient. Unfortunately, small numbers of publicly available full-genomic sequences for GII.4 New Orleans 2009 and Sydney 2012 limited our phylogenetic analysis, and we were unable to include sequences from many geographical regions.

Another common source of variability in RNA viruses is recombination. In this study, we obtained near-full genomic NoV sequences from our samples, which enabled us to perform genome-wide recombination analysis. An intra-genotypic recombination event was observed for BMH16–078, which contains Sydney 2012 ORF2, and New Orleans 2009 ORF1 and ORF3 including the GII.P4 New Orleans 2009 pol gene. This strain circulated in Ontario in 2016. ORF1/2 recombinants of New Orleans 2009 and Sydney 2012 have been previously reported [14, 35, 45]. Additionally, it has been demonstrated that the New Orleans 2009 ORF2 variant has almost disappeared because of recombination with the GII.4 Sydney 2012 ORF2 variant [46]. However, unlike previously reported New Orleans 2009/Sydney 2012 recombinants, the recombination breakpoints of BMH16–078 flank the ORF2, creating a mosaic New Orleans 2009 virus with Sydney 2012 capsid. Due to limited number of full-length GII.4 sequences available, such a sequence has not been reported previously; however, its existence has been inferred from phylogenetic analysis [47]. Comprehensive molecular epidemiological studies are required in order to determine the source and mechanisms facilitating the emergence of this GII.4 variant.

Amino acid substitutions were observed throughout the VP1 protein, but the majority of the changes were located on the outer surface of the P domain, near or at blockade epitopes and conformational epitopes. Further in vivo and in vitro investigations are required in order to validate whether these substitutions alter the antigenic profile of these viruses. None of the studied samples from patients contained a virus that showed changes in the receptor-binding pocket sites, indicating that the receptor specificity was unchanged among these strains. These results further validate that the P domain of the VP1 protein in GII.4 variants is subject to strong selective pressure that may produce immune escape variants while the receptor binding sites remain relatively conserved.

While genomic similarity analysis verifies that the capsid region is quite heterogeneous between members of the same strain, it indicates that the protease and the polymerase genes are relatively conserved. Another study has also reported that these genes can tolerate few nucleotide changes [48]. This observation is not unexpected due to their critical functions in viral replication, viral protease and RdRP genes are highly conserved [40, 49] and have been attractive targets for the design and development of antiviral strategies. Nucleotide diversity was also observed in regions of the p48, NTPase and VPg genes. Certain regions of these genes have been shown to be able to tolerate drastic nucleotide changes [48], and are evolutionarily less conserved [50].

The presence of other pathogens can have a significant effect on the severity and outcome of NoV infection. For proper metagenomics analysis of a microbial community such as intestinal microbiota, shotgun sequencing of DNA and RNA as well as 16S rRNA sequencing should be conducted [51]. As a-proof-of-concept, by performing 16S rRNA gene sequencing, and metagenomic shotgun sequencing, a wide variety of enteric pathogens were identified in diarrhea stool samples [52]. Since stool filtrates were used in this study, the presence of pathogenic enteric bacteria in the samples was not explored. However, shotgun RNA sequencing of stool filtrates enabled us to examine the presence of other enteric RNA viruses. Herein, we identified a number of sequencing reads that mapped to human astrovirus in sample NV-13-0152. Human astrovirus is a major cause of gastroenteritis in children under the age of 5 [53] and this patient was 15 months old at the time of sample collection. It seems that norovirus-astrovirus co-infection is likely to occur in childcare facilities, however due to the lack of epidemiological and metagenomics data, they may have been under reported.

Overall, in this study, limited numbers of prevailing GII.4 strains were characterized and an accumulation of data from molecular epidemiological studies with continuous surveillance are required for developing prediction systems for NoV outbreaks or an efficient vaccine strategy.

The use of NGS for molecular epidemiology advances our understanding regarding the transmission dynamics of NoV and allows for timely interventions and outbreak control practices, thus reducing transmission and decreasing the burden of norovirus infection. In summary, our study provides detailed analyses of the genetic diversity of NoV GII.4 in Canada. Nevertheless, it is important to continue to monitor and characterize circulating NoV strains in real-time to identify emerging variants that can escape from previously acquired immunity and cause epidemics.

## Conclusion

In conclusion, 19 near-full GII.4 genome sequences were retrieved by RNA-Seq method from stool filtrates. The majority of genomes belong to Sydney 2012 strains, while two isolates showed homology to New Orleans 2009 strains. Also, one recombinant sequence was identified. The genomic data were further analyzed for genetic similarity between the isolates as well as identification of non-synonymous changes in the major capsid protein and the viral polymerase protein. Co-infection with other enteric RNA viruses was also investigated.

## Additional file

Additional file 1: **Table S1.** Full-genome sequences obtained by De novo assembly. Viral load is given in genome copies/μl. * Fold coverage refers to the median coverage across the genomes. **Figure S1**. The coverage profile of the sequenced GII.4 variants. Coverage was calculated as the total number of reads covering a given nucleotide and was normalized by the sum of total coverage across the genome. i.e., at each residue, the coverage was divided by the total coverage and the sum of normalized coverage equals one. **Figure S2**. Reads from NV13–0152 were referenced mapped to KF039912.1 (Human astrovirus 4 isolate Rus-Nsc05–623 complete genome).The resulting assembly was inspected with Qualimap [54]. (DOCX 336 kb)

## Abbreviations

NGS: Next-generation Sequencing
NoV: Norovirus
ORF: Open reading frame
RdRp: RNA dependent RNA polymerase
WGS: Whole genome sequencing
