# Supplementary material for: Genetic characterization of norovirus GII.4 variants circulating in Canada using a metagenomic technique
Source: BMC Infect Dis. 2018 Oct 17;18:521. doi: 10.1186/s12879-018-3419-8 (PMC6191920; doi:10.1186/s12879-018-3419-8)
Supplement: Supplementary file 1 — Table S1. Full-genome sequences obtained by De novo assembly. Viral load is given in genome copies/μl. * Fold coverage refers to the median coverage across the genomes. Figure S1. The coverage profile of the sequenced GII.4 variants. Coverage was calculated as the total number of reads covering a given nucleotide and was normalized by the sum of total coverage across the genome. i.e., at each residue, the coverage was divided by the total coverage and the sum of normalized coverage equals one. Figure S2. Reads from NV13–0152 were referenced mapped to KF039912.1 (Human astrovirus 4 isolate Rus-Nsc05–623 complete genome).The resulting assembly was inspected with Qualimap [54]. (DOCX 336 kb) [file 12879_2018_3419_MOESM1_ESM.docx]

**Additional file 1**

**Table S1.** Full-genome sequences obtained by *De novo* assembly. Viral load is given in genome copies/μl. * Fold coverage refers to the median coverage across the genomes

**Figure S1.** The coverage profile of the sequenced GII.4 variants. Coverage was calculated as the total number of reads covering a given nucleotide and was normalized by the sum of total coverage across the genome. i.e., at each residue, the coverage was divided by the total coverage and the sum of normalized coverage equals one.

**Figure S2.** Reads from NV13-0152 were referenced mapped to KF039912.1 (Human astrovirus 4 isolate Rus-Nsc05-623 complete genome).The resulting assembly was inspected with Qualimap [54].

| **Sample ID** | **Viral Load** | **% Viral Reads** | **Fold Coverage** |
| --- | --- | --- | --- |
| BMH15-063 | 1840 | 0.04% | 31 |
| BMH16-078 | 64300 | 13.23% | 3057 |
| NV12-0010 | 40080 | 29.16% | 1313 |
| NV14-0037 | 1924 | 0.23% | 89 |
| NV13-0130 | 1724 | 0.09% | 60 |
| NV13-0137 | 23784 | 1.18% | 1305 |
| NV13-0143 | 4440 | 0.14% | 23 |
| NV13-0149 | 2128 | 0.52% | 48 |
| NV13-0152 | 1960 | 1.08% | 36 |
| NV13-0156 | 1160 | 0.04% | 35 |
| NV13-0162 | 2280 | 0.11% | 99 |
| NV13-0164 | 3520 | 0.75% | 440 |
| NV14-0022 | 1416 | 0.01% | 20 |
| NV14-0037 | 1923 | 0.08% | 50 |
| NV14-0043 | 3864 | 0.60% | 446 |
| NV14-0045 | 1432 | 0.05% | 22 |
| NV14-0057 | 4976 | 0.02% | 20 |
| NV15-0140 | 1648 | 0.16% | 24 |
| NV15-0178 | 756 | 0.01% | 18 |

**Table S1**


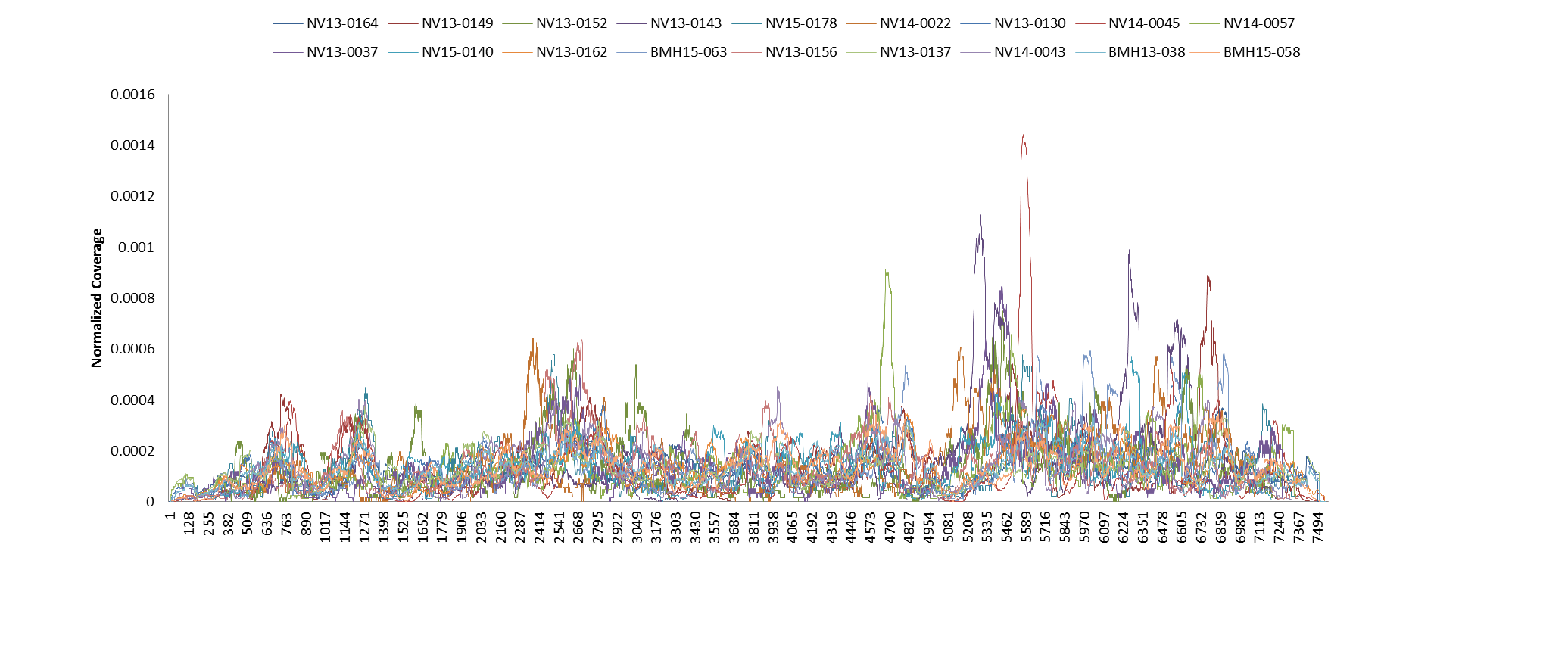


**Figure S1**

**
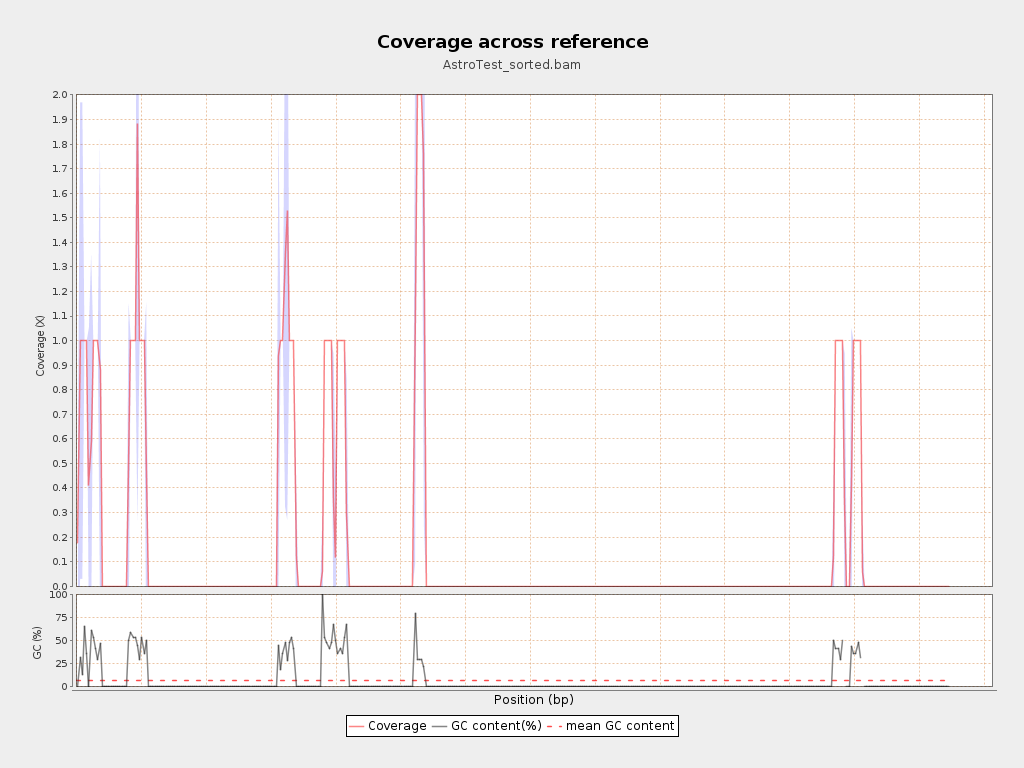
**

**Figure S2**
